# Nerve ultrasound helps to distinguish CIDP patients with diabetes from patients with diabetic polyneuropathy

**DOI:** 10.1038/s41598-024-82235-8

**Published:** 2024-12-16

**Authors:** Bianka Heiling, Katharina Kneer, Winnie He, Thomas Lehmann, Nicolle Müller, Christof Kloos, Alexander Grimm, Hubertus Axer

**Affiliations:** 1 Department of Neurology, Jena University Hospital, Friedrich Schiller University, 07747 Jena, Germany; 2 Clinician Scientist Program OrganAge, Jena University Hospital, 07747 Jena, Germany; 3 Department of Neurology, Tuebingen University Hospital, 72076 Tuebingen, Germany; 4 Center for Clinical Studies, Jena University Hospital, Jena, Germany; 5 Department of Internal Medicine III, Jena University Hospital, Friedrich Schiller University, 07747 Jena, Germany

**Keywords:** Peripheral nervous system, Peripheral neuropathies

## Abstract

Diabetic polyneuropathy (DPN) shares overlapping clinical and electrodiagnostic features with chronic inflammatory demyelinating polyneuropathy (CIDP), which complicates the differential diagnosis of CIDP in diabetic patients. 32 patients with diabetes mellitus and CIDP, 68 patients with CIDP without diabetes, 83 patients with DPN, and 28 diabetic patients without polyneuropathy were examined using clinical scores (Overall Neuropathy Limitation Scale (ONLS), Neuropathy Symptom Score, Neuropathy Deficit Score), nerve conduction studies, and nerve ultrasound (Ultrasound Pattern Sum Score (UPSS)). The ONLS was significantly higher in the CIDP patients with diabetes than in DPN (median [interquartile range]: 4.0 [3.0] vs. 0 [1.0], *p* < 0.001) as well as the UPSS (4.0 [6.0] vs. 0 [2.9], *p* < 0.001). Multiple binary logistic regression revealed UPSS and ONLS as statistically significant predictors to differentiate between CIDP with diabetes and DPN. Receiver operating characteristic curve analysis showed the ONLS with an area under the curve (AUC) of 0.918 (95% CI: 0.868-0.0.967, *p* < 0.001). The UPSS total score had an AUC of 0.826 (95% CI: 0.743–0.909, *p* < 0.001). An UPSS ≥ 2.5 had a sensitivity of 77.4% and a specificity of 68.7% to detect CIDP. An ONLS ≥ 1.5 had a sensitivity of 87.1% and a specificity of 81.9% to detect CIDP. ROC curve analysis of a composite score of ONLS and UPSS revealed an AUC of 0.959 (95% CI: 0.928–0.991, *p* < 0.001). CIDP is an important differential diagnosis in people with diabetes mellitus. This study reports that the UPSS is well suited to differentiate between DPN and CIDP.

**Supplementary Information:**

The online version contains supplementary material available at 10.1038/s41598-024-82235-8.

## Introduction

Diabetic polyneuropathy (DPN) is a significant complication of diabetes mellitus^1^ with a prevalence reaching from 10% in patients with type 2 diabetes increasing to about 50% after 10 or more years of diabetes duration^2^. Differentiation from neuropathies of other etiologies can be difficult. In particular, chronic inflammatory demyelinating polyneuropathy (CIDP) is often associated with diabetes mellitus^3^. The differentiation between both entities is important especially regarding their different way of treatment. In general, the current literature is incoherent regarding the overall increased risk for diabetic patients to develop CIDP: whereas some studies describe an increased risk by 9 to 11-fold^3–5^, some studies describe no increase in disease prevalence^6–8^.

The most common type of DPN is a distal symmetric polyneuropathy^5^, which is clinically characterized by distally and symmetrically distributed abnormal sensation, pain, paresthesia, and weakness. Nerve conduction studies (NCS) often show an axonal (i.e. reductions in amplitude, slight conduction slowing) or axonal-demyelinating neuropathy, but there are also a number of patients with a predominantly demyelinating pattern (marked conduction slowing, conduction blocks, temporal dispersion), which can be difficult to distinguish from CIDP^9^.

High resolution ultrasound of peripheral nerves has been proven to provide additional diagnostic information on several polyneuropathies^10^. Padua et al. were one of the first to describe nerve enlargement in patients with CIDP^11^. Nerve enlargement in CIDP is heterogeneous, but mostly regionally to focally restricted with distinct echointensity pattern. In general, demyelinating neuropathies are more often associated with nerve enlargement than axonal neuropathies^12,13^.

Nerve ultrasound results in DPN have been heterogeneous, and nerve enlargement was, in comparison to CIDP patients, more subtle. A number of authors describe nerve enlargement in patients with type 2 diabetes^14–19^, type 1 diabetes^16,19,20^, or in mixed diabetes types^21–24^ suffering from polyneuropathy, whereas others did not find any nerve enlargement^25,26^.

Overall, the detection of CIDP in diabetic individuals is of major importance since the risk to develop CIDP may be increased in people with diabetes mellitus and prompt treatment of CIDP should be initiated in order to prevent progressive neurological deficits. The differentiation of DPN from CIDP is challenging because both may share similar clinical and electrophysiological features. Severe neuropathy despite good glycemic control, fast deterioration of polyneuropathy, less pain, and prolonged F-wave latency, reduced conduction velocity, conduction block and temporal dispersion in NCS may be criteria to favor CIDP diagnosis over DPN^27,28^.

As peripheral nerve ultrasound shows nerve swelling in inflammatory neuropathy it may be useful in the differentiation between CIDP and DPN. Therefore, we compared clinical parameters, NCS, and nerve ultrasound of individuals with DPN to CIDP patients with diabetes (and without diabetes) to identify parameters suitable for a reliable detection of CIDP in diabetic patients.

## Methods

### Patients

Data of CIDP patients were retrospectively collected from patients who were referred to the Department of Neurology at Jena University Hospital or to the Neuromuscular Center at University Hospital Tübingen between 2018 and 2021. The study was approved by the local ethics committees (Jena: project number 2022–2790 Daten, Tübingen: project number 099/2022BO2). We used the STARD reporting guidelines when writing our paper.

As the CIDP patients were collected between 2018 and 2021 the diagnosis was determined according to the diagnostic criteria of the Joint Task Force of the European Federation of Neurological Societies and the Peripheral Nerve Society (EFNS/PNS) from 2010^29^. CIDP diagnosis was based on the clinical course, electrodiagnostic criteria, and cerebrospinal fluid examination.

Cases with the final clinical diagnosis of CIDP were included in the study. Asymmetric variants of CIDP and other inflammatory polyneuropathies (e.g. multifocal acquired demyelinating sensory and motor neuropathy (MADSAM), multifocal motor neuropathy (MMN), chronic idiopathic axonal polyneuropathy (CIAP), or vasculitic neuropathy), as well as neuropathy due to other causes such as toxic, critical illness, or hereditary polyneuropathy were excluded from the study. The included CIDP patients were subdivided into a group with (= group 2) and without diabetes mellitus (= group 1).

In addition, we analyzed a database of people with type 2 diabetes mellitus recruited in the SELECT study, which has been registered in the German clinical trial register (DRKS00023026) and approved by the local ethics committee (Jena: project number 2019-1416-BO). These data were prospectively collected between September 2020 and January 2023. The cohort comprises individuals with type 2 diabetes mellitus with and without DPN, aged between 40 and 85 years, who signed informed consent, willing to undergo NCS and nerve ultrasound and able to complete questionnaires. Patients with other known etiology for polyneuropathy such as rheumatic disease, peripheral arterial occlusive disease, active malignant tumor disease, and history of chemotherapy were excluded from the study. Patients with peripheral arterial occlusive disease were excluded because it is associated with axonal damage^30^ and it often leads to amputations or open wounds, which impede NCS measurements. The patients were divided in two subgroups: participants with diabetes mellitus without polyneuropathy (= group 3) and with DPN (= group 4).

### Assessments

We collected baseline characteristics such as age, gender, body mass index (kg/m^2^), as well as HbA1c (%) and glomerular filtration rate (ml/min).

Physical limitations due to polyneuropathy were evaluated using the Overall Neuropathy Limitation Scale (ONLS)^31^. The arm scale asks for symptoms in hands or arms (tingling, numbness, weakness) and fine motor skills (dressing the upper body, washing/combing hair, turning a key in a lock, using a knife and fork together, do or undo buttons/zips). The leg score asks for difficulties in walking, running or climbing stairs, abnormal gait, mobility for 10 m, and the need for a walking aid. The arms score grading from 0 to 5 and the legs score grading from 0 to 7 add up to a total score ranging from 0, given for no symptoms, to 12 points, given for patients without purposeful movements in both arms and legs.

The Neuropathy Symptom Score (NSS) and the Neuropathy Deficit Score (NDS) are often used to detect neuropathic symptoms in diabetic patients^32^. The NSS categorizes the severity of sensory symptoms by asking for symptoms in feet and legs (like burning, numbness, tingling, fatigue, cramping, aching), the localization and patterns of appearance and improvement of symptoms^33^. The NDS scores the ankle reflex status, the perception threshold of vibration using a tuning fork, pin-prick and temperature sensation^33^. In this study, the scales of NSS and NDS were used to assess severity with a focus on sensory symptoms.

### Nerve ultrasound

In the CIDP cohort (group 1 and 2) ultrasound studies of peripheral nerves were performed with an 18 or 24 MHz high-resolution probe (Canon Aplio i800, Canon Medical Systems USA, Inc. 2441 Michelle Drive Tustin, CA 92780) by three experienced neurologists. In the diabetes patients (group 3 and 4) ultrasound measurements were executed by a single experienced neurologist using a high-resolution ultrasound device (Mindray M7, Medical Australia Ltd., Ultrasound systems, Darmstadt, Germany) with a 14 MHz linear-array transducer.

The cross-sectional area (CSA) of predefined nerves was measured by tracing the inner border of the hyperechoic rim of each nerve at predefined landmarks. The horizontal diameter of the cervical nerve roots was measured within the hyperechoic rim perpendicular to their course. The Ultrasound Pattern Sum Score (UPSS, Table 1)^34,35^ was used to grade the ultrasound measurements. The anatomical locations of the nerve segments that were scanned were strictly defined and can be seen in Table S1.

### Nerve conduction studies

In both patient groups nerve conduction studies (NCS) were performed by experienced neurological staff using a Medelec Synergy device (Synergy 15.0; Viasys Healthcare, Natus Europe GmbH, Planegg, Germany). Measurements were executed on the median, tibial and sural nerve. On the median and tibial nerve distal motor latency (DML), motor nerve conduction velocity (MCV), amplitudes of compound muscle action potential (CMAP) as well as F-wave response were determined. Sensory nerve conduction studies were done on the median and sural nerve measuring the amplitude of sensory nerve action potential (SNAP) and sensory nerve conduction velocity (SCV). Skin temperature was controlled to be between 32 and 34 °C.

Distal symmetric polyneuropathy was defined according to the AAEM (American Association of Electrodiagnostic Medicine) criteria, which state that an abnormality of any attribute of nerve conduction has to be found in at least two separate nerves, one of which must be the sural nerve^36^.

### Statistics

All data were analyzed using the Statistical Package for the Social Sciences software (SPSS version 29.0, IBM Corporation, Armonk, NY, USA). Continuous variables were summarized by median and interquartile range (IQR) and categorical variables by absolute and relative frequencies. To assess differences between the four different patient groups we applied the non-parametric Kruskal-Wallis test for continuous data, and the Mann-Whitney U test was performed to compare continuous data between two groups. P values were adjusted using Bonferroni correction for multiple tests. Fisher’s exact test was used for detecting differences in rates of detected enlarged nerves between the groups. Multiple binary logistic regression analysis was used to evaluate predictors to differentiate between the patients with CIDP and diabetes and the patients with DPN. For all analyses, a p value < 0.05 was considered statistically significant. Receiver operating characteristic (ROC) curve analysis was performed to evaluate the use of different parameters to distinguish CIDP patients with diabetes from patients with DPN. The overall precision of the diagnosis is given by the area under the curve (AUC) with 95% confidence interval (CI). An AUC of 1 represents highest accuracy, whereas an AUC of 0.5 represents purely random decisions independent from the measurements with no diagnostic value of the test. Highest Youden-Index was used to determine cut-offs of the scores, and sensitivity as well as specificity are presented to evaluate the accuracy of predictions.

## Results

### Patients

100 patients with CIDP and 111 patients with diabetes type 2 were included in the study. 32 patients of the CIDP group also had type 2 diabetes mellitus (group 2), 68 had no diabetes mellitus (group 1). Of the included patients with type 2 diabetes, 83 patients presented with DPN (group 4) and 28 had no polyneuropathy (group 3).

Table 2 shows the baseline characteristics as well as the clinical scores of all patients. The patients of the individual groups did not differ predominantly in age; only the diabetic patients without polyneuropathy (median [IQR]: 62.0 years [17.5], *p* = 0.048) as well as the CIDP patients without diabetes (61.0 [26.3], *p* = 0.02) were significantly younger than the patients with diabetic polyneuropathy (68.0 [14.0]).

HbA1c was significantly lower in the CIDP group without diabetes (5.4% [0.5]), compared to all other groups (CIDP with diabetes: 7.1% [1.6], diabetes without neuropathy: 7.5% [1.9], diabetes with DPN: 7.8% [1.0], *p* < 0.001 each). Glomerular filtration rate (GFR) was unimpaired in the CIDP group without diabetes (GFR 96.5 ml/min [32.1]) and slightly impaired in all other groups (CIDP with diabetes: 76.5 [33.2], diabetes without neuropathy: 75.3 [37.0], diabetes with DPN: 77.2 [31.3], *p* < 0.001 each).

Body mass index (BMI) did not differ between both CIDP groups. However, BMI values of CIDP patients without diabetes (27.0 kg/m^2^ [7.0]) were significantly lower than all groups with diabetes (CIDP with diabetes: 29.2 [6.0], *p* = 0.017, diabetes without neuropathy: 32.6 [10.0], *p* < 0.001, diabetes with DPN: 32.0 [10.0], *p* < 0.001).

Diabetes duration was significantly longer in diabetic patients with DPN (16.4 years [14.3]) than in diabetic patients without neuropathy (10.2 years [15.1], *p* = 0.023) and in CIDP patients with diabetes (5.5 years [7.6], *p* < 0.001).

### Clinical scores

**Fig. 1:**
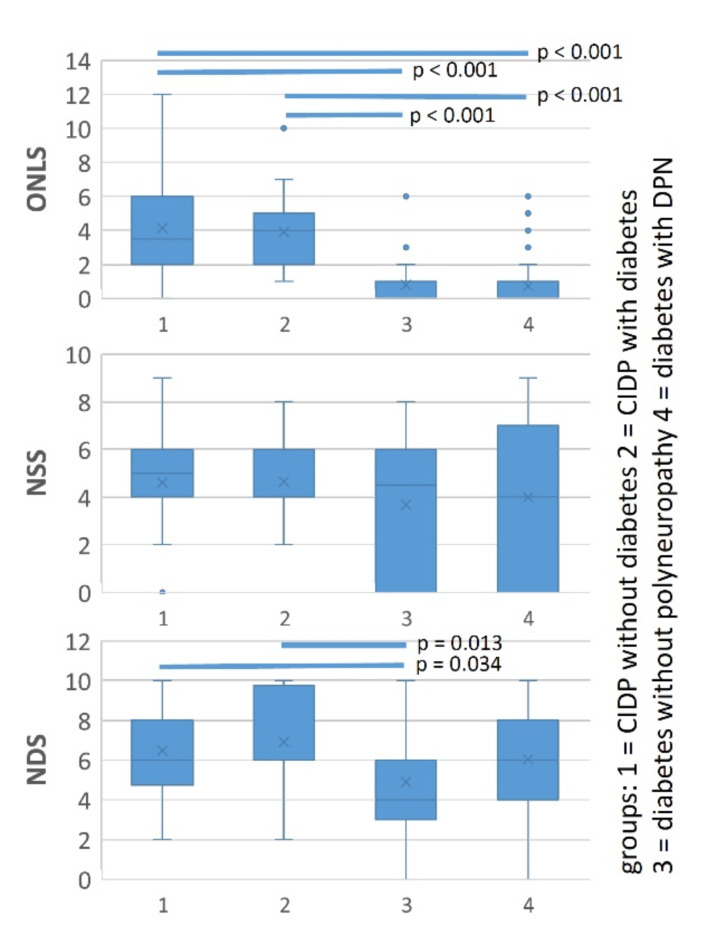
Boxplots of NSS, NDS, and ONLS of all patient groups. Statistically significant differences between groups are shown as crosslines. The ONLS showed significant differences between CIDP patients and diabetes patients (CIDP patients: 4.0 [4.0] vs. diabetes patients: 0 [1.0], *p* < 0.001). Kruskal-Wallis test revealed no differences of the NSS between all groups (*p* = 0.944). The NDS showed differences between patients without DPN and both CIDP groups (CIDP without diabetes: 6.0 [4.0], *p* = 0.034, CIDP with diabetes: 6.0 [3.0], *p* = 0.013). Abbreviations: ONLS = Overall neuropathy limitations scale, NSS = Neuropathy symptom score, NDS = Neuropathy deficit score, CIDP = Chronic inflammatory demyelinating polyneuropathy, DPN = Diabetic polyneuropathy.

Figure 1 shows the distribution of NSS, NDS, and ONLS in the four groups. Statistical analysis using the Kruskal-Wallis test revealed no differences of the NSS between all groups (*p* = 0.944). The NDS showed no differences between CIDP patients with diabetes (6.0 [4.09]) and DPN patients (6.0 [4.0], *p* = 0.648). The diabetic patients without DPN had the lowest NDS (4.0 [3.0]) compared to all other groups (CIDP without diabetes: 6.0 [4.0], *p* = 0.034, CIDP with diabetes: 6.0 [3.0], *p* = 0.013, diabetes with DPN: 6.0 [4.0], *p* = 0.17).

The ONLS showed significant differences between the CIDP patients and the diabetes patients (CIDP patients: 4.0 [4.0] vs. diabetes patients: 0 [1.0], *p* < 0.001). This difference was also statistically significant in the CIDP patients with diabetes compared to the patients with diabetic polyneuropathy (4.0 [3.0] vs. 0 [1.0], *p* < 0.001). Thus, the group of CIDP patients (with or without diabetes) had significantly more severe functional deficits than the patients with DPN.

### Nerve ultrasound

Table 3 shows the ultrasound CSA measurements in the four patient groups. Table 4 shows the relative frequencies of CSA enlargements in the groups. The CSA measurement was regarded to be enlarged, if the CSA exceeded the upper boundary limit of the measured nerve segment as defined in the UPSS^35^. Therefore, we included in Table 4 only those nerve segments, which are part of the UPSS, because the UPSS defines clear-cut boundary values to decide if a nerve segment may be enlarged or not.

Both CIDP groups (CIDP with and CIDP without diabetes) generally showed most often enlargements at the different anatomical landmarks of the peripheral nerves, and nerve enlargements were most often found in the proximal median nerve, the cervical nerve roots and the tibial nerve. Enlargements of the median nerve at the upper arm were found in 61.8% of the patients with CIDP without diabetes and in 56.3% of the patients with CIDP with diabetes. The median nerve at the elbow was enlarged in 52.9% of the patients with CIDP without diabetes and in 59.4% of the patients with CIDP with diabetes. Enlarged C6 nerve root diameters were found in 50.0% of the patients with CIDP without diabetes and in 37.5% of the patients with CIDP with diabetes. Enlarged C5 nerve root diameters were found in 52.9% of the patients with CIDP without diabetes and in 31.3% of the patients with CIDP with diabetes. The tibial nerve at the popliteal fossa was enlarged in 48.5% of the patients with CIDP without diabetes an in 68.8% of the patients with CIDP with diabetes. Enlargements of the tibial nerve at the ankle were detected in 52.9% of the patients with CIDP without diabetes and in 50.0% of the patients with CIDP with diabetes.

In patients with DPN the CSA of the median nerve at the upper arm (25.3%) and at the elbow (30.1%), the CSA of the tibial nerve at the popliteal fossa (20.5%) and the diameter of the C6 nerve root (22.9%) were most often enlarged. The diameter of the C5 nerve root was enlarged in 13.3% of the patients with DPN. The CSA of the tibial nerve at the popliteal fossa was enlarged in 20.5% of the DPN patients and at the ankle in 28.9% of the DPN patients.

Patients with CIDP and diabetes showed statistically higher scores of the UPSS (4.0 [6.0]), the UPSA (3.0 [3.0]), and UPSB (1.0 [2.0]] compared to the patients with DPN (UPSS 1.0 [2.9], *p* < 0.001, UPSA 1.0 [2.0], *p* < 0.001, UPSB 0 [1.0], *p* = 0.005).

### Nerve conduction studies

Table 5 shows the results of nerve conduction studies of all four groups. A considerable number of the nerves were electrically not excitable. In these cases amplitudes were set to 0, but latencies and nerve conduction velocities could not be determined. The missing values are reported separately in Table 5.

CIDP patients with diabetes had significantly larger reductions in distal CMAP amplitude of the tibial nerve (1.0 mV [3.7] vs. 6.7 [8.2], *p* < 0.001), in the MCV of the median nerve (43.6 m/s [11.2] vs. 50.0 [6.0], *p* < 0.001), in the SNAP amplitude of the median nerve (0.9 µV [7.7] vs. 10.4 [8.9], *p* < 0.001), and larger delay in F-wave latency of the median nerve (32.7 ms [6.1] vs. 29.2 [3.7], *p* = 0.007) compared with diabetic patients with polyneuropathy.

### Differentiation between CIDP patients with diabetes and patients with DPN

The clinically important goal is to distinguish diabetic patients with DPN from those with CIDP. Therefore, group 2 has to be distinguished from group 4. Table 6 shows the multiple results of the binary logistic regression, which shows the UPSS (OR = 0.56, 95% confidence interval (CI): 0.34–0.91, *p* = 0.019) and the ONLS (OR = 0.38, 95% CI: 0.22–0.64), *p* < 0.001) as statistically significant predictors to differentiate between these two groups.

**Fig. 2:**
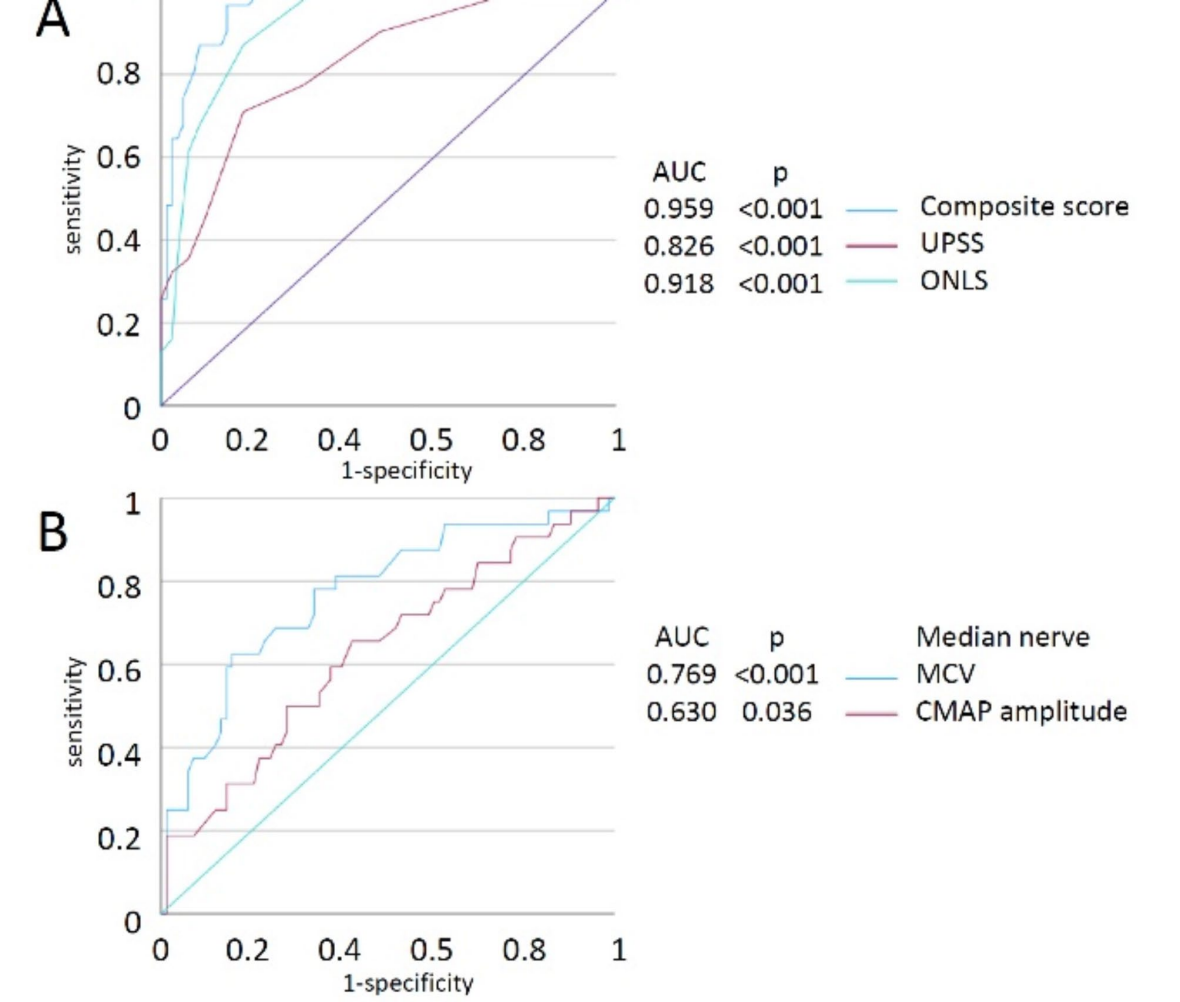
ROC curves. **(A)** UPSS, ONLS and composite score. Figure 2A shows the ROC curves of ONLS and UPSS. The ONLS as a parameter for clinical impairment had an AUC of 0.918 (95% CI: 0.868-0.0.967, *p* < 0.001). The UPSS total score as a comprehensive parameter of nerve enlargement had an AUC of 0.826 (95% CI: 0.743–0.909, *p* < 0.001). An UPSS ≥ 2.5 had a sensitivity of 77.4% and a specificity of 68.7% to detect CIDP. An ONLS ≥ 1.5 had a sensitivity of 87.1% and a specificity of 81.9% to detect CIDP. ROC curve analysis of the composite score demonstrated an AUC of 0.959 (95% CI: 0.928–0.991 *p* < 0.001). A composite score ≥ 0.1292 had a sensitivity of 96.8% and a specificity of 80.7% to detect CIDP. **(B)** Nerve conductions studies of the median nerve. Using NCS measurements of the median nerve AUC was 0.769 (95% CI: 0.669–0.869, *p* < 0.001) for the MCV of the median nerve and 0.630 (95% CI: 0.514–0.745, *p* = 0.032) for the CMAP amplitude of the median nerve. Abbreviations: AUC = area under the curve, UPSS = Ultrasound pattern sum score, ONLS = Overall neuropathy limitations scale, MCV = Motor conduction velocity, CMAP = compound muscle action potential.

Figure 2A shows the ROC curves of ONLS and UPSS. The ONLS as a parameter for clinical impairment had an AUC of 0.918 (95% CI: 0.868-0.0.967, *p* < 0.001). The UPSS total score as a comprehensive parameter of nerve enlargement had an AUC of 0.826 (95% CI: 0.743–0.909, *p* < 0.001). An UPSS ≥ 2.5 had a sensitivity of 77.4% and a specificity of 68.7% to detect CIDP. An ONLS ≥ 1.5 had a sensitivity of 87.1% and a specificity of 81.9% to detect CIDP.

In contrast, using NCS measurements of the median nerve AUC was 0.769 (95% CI: 0.669–0.869, *p* < 0.001) for the MCV of the median nerve and 0.630 (95% CI: 0.514–0.745, *p* = 0.032) for the CMAP amplitude of the median nerve (Fig. 2B).

In order to enhance the diagnostic performance UPSS and ONLS were normalized and combined in a composite score with a range from 0 to 1:

composite score = ((UPSS / 20) + (ONLS / 12)) / 2.

ROC curve analysis of the composite score demonstrated an AUC of 0.959 (95% CI: 0.928–0.991 *p* < 0.001). A composite score ≥ 0.1292 had a sensitivity of 96.8% and a specificity of 80.7% to detect CIDP.

## Discussion

A large database study encompassing 101,321,694 people showed a prevalence of CIDP of 0.008%, a prevalence of diabetes mellitus of 4%, a prevalence of CIDP without diabetes mellitus of 0.006%, and a prevalence of CIDP with diabetes mellitus of 0.054%^3^. Although the association of CIDP and diabetes mellitus has been a matter of controversy^37^, recent studies showed a relatively large number of patients with CIDP also suffering from diabetes mellitus. Data from the Italian CIDP Database study group showed 14% of 393 patients with CIDP to also have diabetes^38^. In addition, 18% of 139 patients of patients with CIDP had diabetes in a Serbian cohort, 19% of 114 patients in a UK cohort^39^, and 18% of 134 patients in a Japanese cohort^37^.

These data show an overall increased risk of co-occurrence of diabetes mellitus and CIDP, the diagnosis of CIDP in diabetic patients remains difficult in clinical everyday life. Red flags for diabetic patients suffering from neuropathic symptoms suggesting CIDP instead of DPN include severe neuropathy despite good glycemic control^27^, fast deterioration of polyneuropathy and prevalent demyelinating features in NCS^28^.

The CIDP patients in our study were retrospectively collected in two tertiary care neuromuscular centers. Diagnostic criteria used included the clinical course, electrodiagnostic criteria as recommended^29^, as well as cerebrospinal fluid analysis showing cytoalbuminologic dissociation in CIDP patients (see Table 2).

Our cohort included 32 CIDP patients with a past medical history of diabetes mellitus, which seems to be higher than in other studies described^6,7^. However, prevalence of diabetes mellitus in Germany in the age group from 65 to 79 years is 23.9%^40^ and our study was not designed as an epidemiologic inquiry. Nevertheless, the co-occurrence demonstrates the necessity to define robust criteria to distinguish CIDP from DPN.

Although NCS measurements showed the CIDP cohorts having more demyelinating changes than the diabetic cohorts, ROC curve analysis showed that a differentiation between DPN and CIDP in diabetic patients can be done more precisely by using the ultrasound measurements (represented by the UPSS).

As DPN is not strictly limited to an axonal pattern, but may also present with a mixed axonal-demyelinating and a predominantly demyelinating pattern^9^, an interpretation using amplitudes and conduction velocity alone may not be sensitive and specific enough even in severely affected patients, in which electrical signals of peripheral nerves may not be evocable at all. Further, in the CIDP patients with diabetes a mixed pathophysiology of inflammatory and diabetic neuropathy may be concurrently present.

Lotan et al.^28^ suggested a total score including supportive and contradictive clinical indicators, nerve conduction studies and paraclinical parameters to be used for diabetic patients when screening for CIDP and allowing a further stratification into sub-categories, such as unlike, possible, probable, or definite CIDP cases.

Clinical severity of polyneuropathy was a well-suited parameter to distinguish both groups. Especially the ONLS exhibits a good AUC in ROC curve analysis. NSS and NDS are routinely used and are widely propagated to screen for diabetic neuropathy^32^, but the scores performed insufficiently to differentiate CIDP from DPN. Especially the assessment of functional impairments of daily living, screened by the ONLS, hosts an advantage when compared to NSS and NDS, evaluating sensory symptoms and clinical signs of neuropathy.

However, the use of disability scales to separate CIDP from diabetic neuropathy may be inappropriate. It is known that CIDP generally produces more severe disability over time^28^. Therefore, differences in duration of illness between the groups, potentially contributions of co-morbidities, and minimally disabling forms of CIDP may make disability alone a precarious criterion in clinical routine.

The UPSS was the second parameter, which also allowed to differentiate diabetic CIDP patients from patients with DPN. Tan et al.^41^ compared nerve ultrasound of 9 diabetic patients with predominantly demyelinating DPN and 10 diabetic patients with CIDP and found larger nerves at the proximal and non-entrapment sites of the upper limbs in the CIDP patients. However, this study is limited by a small sample size.

We used the UPSS as a comprehensive ultrasound score to further stratify our patient cohort. The score itself proved to be a reliable tool to further differentiate neuropathies in general, especially inflammatory from non-inflammatory neuropathies. Hereby, a score of > 4 points indicates a possible inflammatory cause of symptoms^25,42^.

Nerve ultrasound in CIDP patients shows a heterogeneous enlargement of nerves and typically an UPSS of more than 5 points^43^. In contrast, DPN patients tend to present with slight nerve enlargement, especially at entrapment sites with an UPSS of less than 3 points^43^. Therefore, it is quite evident to use nerve ultrasound to differentiate between these two entities.

The second revision of the European Academy of Neurology/Peripheral Nerve Society guideline on diagnosis and treatment of CIDP^44^ implemented ultrasound criteria for the first time as supportive criteria for CIDP diagnosis. CIDP diagnosis is supported if nerve enlargement of at least two sites in proximal median nerve segments and/or the brachial plexus and nerve roots are detected^44^. The CIDP patients in our study showed frequent CSA enlargements in the proximal parts of the median nerve as well as in the cervical nerve roots (see Table 4). However, CSA enlargements in these regions were also present in patients with DPN, but less frequent and less pronounced. To increase diagnostic accuracy, it is therefore necessary to examine more nerves with ultrasound in order to discriminate CIDP from DPN. The use of the UPSS at this point is easy, strikingly effective and clear.

Some limitations of the study need to be discussed. Data on diabetic patients and CIDP patients were not collected in the same study and different ultrasound probes were used, which may cause some systematic bias. Due to the retrospective character of the CIDP data, inter-equipment reliability measurements were not possible, and may therefore be a significant limitation of our study. In addition, intrarater and interrater ICC (intraclass correlation coefficient) were not assessed.

However, patient numbers were relatively high, and the results for NCS and ultrasound in the groups were sound in comparison to the literature. The duration of CIDP or diabetes was not controlled in our patients and most of the patients were not therapy-naïve. This may have some impact on ultrasound results^45^. Other parameters, such as cerebrospinal fluid analysis or nerve biopsy results have not been done in the work-up of the DPN patients.

In summary, this study reports that UPSS is well suited to differentiate between diabetic patients with DPN and diabetic patients with CIDP. This may provide important information to facilitate the differential diagnosis of CIDP or to promote further medical tests such as cerebrospinal fluid analysis or nerve biopsy. This is of major importance as inflammatory neuropathies such as CIDP should be treated with anti-inflammatory measures^44^, such as immunoglobulin, glucocorticoids, or plasmapheresis to prevent progressive decline of peripheral nerve function. In addition, it is important to propagate the information to general practitioners treating people with diabetes mellitus that rapid loss of function in people with often short diabetes duration is uncommon for DPN and should lead to neurological referral.

**Table 1:** The UPSS is the sum of the three sub-scores and can reach from 0 to 20 points.

Subscores	Unilateral measurements	Scoring	Range
UPSA	CSA of median nerve at the upper arm, elbow, and mid-forearm CSA of ulnar nerve at the upper arm and mid-forearm CSA of tibial nerve popliteal and at the ankle CSA of fibular nerve popliteal.	Each nerve enlargement < 50% of the defined maximum values is assigned 1 point and each enlargement > 50% is scored with 2 points	0–16 points
UPSB	diameter of the 5th and 6th cervical nerve root CSA of vagus nerve at mid-neck level	Each enlargement is assigned with 1 point	0–3 points
UPSC	CSA of sural nerve	Enlargement is assigned with 1 point	0–1 point

**Table 2:** Baseline characteristics of CIDP patients (*n* = 100) and diabetes patients (*n* = 111). A p value < 0.05 was considered statistically significant.

Patient characteristics	Group 1 (CIDP without diabetes)	Group 2 (CIDP with diabetes)	Group 3 (Diabetes without neuropathy)	Group 4 (Diabetes with DPN)	Mann-Whitney U test group 2 vs. group 4
	68	32	28	83	
Age, years (IQR)	61.0 (26.3)	68.5 (16.8)	62.0 (17.5)	68.0 (14.0)	*p* = 1.000
Male gender, n (%)	50 (73.5)	25 (78.1)	12 (42.9)	55 (66.3)	
Height, m (IQR)	1.78 (0.16)	1.75 (0.13)	1.675 (0.11)	1.73 (0.13)	*p* = 1.000
HbA1c, % (IQR)	5.4 (0.5)	7.1 (1.6)	7.5 (1.9)	7.8 (1.0)	*p* = 0.439
GFR, ml/min (IQR)	96.5 (32.1)	76.5 (33.2)	75.3 (37.0)	77.2 (31.3)	*p* = 1.000
BMI, kg/m² (IQR)	27.0 (7.0)	29.2 (6.0)	32.6 (10.0)	32.0 (10.0)	*p* = 0.967
Diabetes duration, years (IQR)	-	5.5 (7.6)	10.2 (15.1)	16.4 (14.3)	*p* < 0.001
CIDP duration, years (IQR)	5.0 (7.28)	1.2 (7.0)	-	-	
CSF protein, mg/l (IQR)	724.0 (445.0)	800.0 (350.0)	-	-	
CSF cell count, /µl (IQR)	1.0 (1.0)	2.0 (2.0)	-	-	
ONLS (IQR)	3.5 (4.0)	4.0 (3.0)	0 (1.0)	0 (1.0)	*p* < 0.001
NSS (IQR)	5.0 (2.0)	4.0 (2.0)	4.5 (6.0)	4.0 (7.0)	*p* = 1.000
NDS (IQR)	6.0 (3.0)	6.0 (4.0)	4.0 (3.0)	6.0 (4.0)	*p* = 0.791

**Table 3:** Ultrasound data of the different patient groups. A p value < 0.05 was considered statistically significant.

Ultrasound data		Group 1 (CIDP without diabetes)	Group 2 (CIDP with diabetes)	Group 3 (Diabetes without neuropathy)	Group 4 (Diabetes with DPN)	Mann-Whitney U test group 2 vs. group 4
Nerve CSA mm² (IQR)		68	32	28	83	
Median	Upper Arm	12.8 (6.3)	12.0 (4.9)	9.0 (3.0)	10.0 (4.0)	*p* = 0.001
	Elbow	12.0 (6.5)	12.7 (4.9)	9.5 (3.0)	9.0 (4.0)	*p* = 0.003
	Forearm	8.7 (4.8)	8.6 (4.2)	6.0 (2.0)	7.0 (3.0)	*p* = 0.001
	Wrist	12.0 (4.7)	11.5 (6.6)	11.5 (5.5)	13.0 (5.3)	*p* = 0.897
Ulnar	Upper Arm	8.5 (5.0)	8.0 (3.4)	6.0 (2.0)	6.0 (2.0)	*p* < 0.001
	Elbow	11.0 (5.2)	10.4 (4.1)	9.0 (4.8)	10.0 (5.0)	*p* = 1.000
	Forearm	7.0 (3.9)	6.7 (3.0)	5.0 (2.0)	5.0 (2.0)	*p* < 0.001
	Wrist	6.2 (3.0)	6.0 (1.8)	5.0 (1.0)	6.0 (2.0)	*p* = 1.000
Tibial	Popliteal	32.1 (13.6)	37.5 (14.9)	24.0 (9.5)	24.0 (10.5)	*p* < 0.001
	Tarsal Tunnel	15.0 (8.5)	14.4 (6.0)	11.5 (3.8)	12.0 (5.0)	*p* = 0.048
Fibular	Popliteal	10.0 (6.0)	8.9 (4.2)	5.0 (2.0)	6.0 (2.3)	*p* < 0.001
	Fibular Head	11.5 (4.4)	12.9 (8.0)	10.0 (3.0)	10.0 (5.0)	*p* = 0.967
Sural	Calf	3.0 (2.0)	3.0 (1.7)	3.0 (1.0)	2.0 (1.0)	*p* = 0.028
Median nerve root Diameter mm (IQR)						
Vagus	Mid-neck	3.0 (1.8)	3.0 (2.0)	2.5 (1.0)	2.0 (1.0)	*p* = 0.297
C5		3.1 (1.0)	2.9 (0.6)	2.2 (0.8)	2.4 (0.6)	*p* < 0.001
C6		4.3 (1.3)	4.1 (1.0)	3.5 (0.8)	3.6 (1.0)	*p* = 0.014
Median UPSS score (IQR)						
UPSA		4.0 (5.0)	3.0 (3.0)	1.0 (1.0)	1.0 (2.0)	*p* < 0.001
UPSB		1.0 (1.0)	1.0 (2.0)	0 (1.0)	0 (1.0)	*p* = 0.005
UPSC		0 (1.0)	0 (1.0)	0 (0)	0 (0)	*p* = 0.089
UPSS total score		6.0 (7.0)	4.0 (6.0)	1.0 (1.0)	1.0 (2.0)	*p* < 0.001

**Table 4:** Relative frequencies of detected nerve enlargements according to the UPSS. Note that diabetic patients with CIDP had statistically significant more nerve enlargements in median, ulnar, tibial, and fibular nerves than patients with DPN. A p value < 0.05 was considered statistically significant.

		Group 1 (CIDP without diabetes)	Group 2 (CIDP with diabetes)	Group 3 (Diabetes without neuropathy)	Group 4 (Diabetes with DPN)	Fisher’s exact test group 2 vs. group 4
Nerve	Localisation	68	32	28	83	
		Relative frequencies (Number)				
Median	Upper arm	61.8% (42)	56.3% (18)	17.9% (5)	25.3% (21)	*p* = 0.004
	Elbow	52.9% (36)	59.4% (19)	17.9% (5)	30.1% (25)	*p* = 0.005
	Forearm	38.2% (26)	34.4% (11)	7.1% (2)	12.0% (10)	*p* = 0.013
Ulnar	Upper arm	38.2% (26)	28.1% (9)	0.0% (0)	6.0% (5)	*p* = 0.003
	Forearm	30.9% (21)	25.0% (8)	0.0% (0)	2.4% (2)	*p* < 0.001
Tibial	Popliteal	48.5% (33)	68.8% (22)	7.1% (2)	20.5% (17)	*p* < 0.001
	Ankle	52.9% (36)	50.0% (16)	21.4% (6)	28.9% (24)	*p* = 0.029
Fibular	Popliteal	39.7% (27)	31.3% (10)	3.6% (1)	3.6% (3)	*p* < 0.001
Vagus	Mid-neck	25.0% (17)	25.0% (8)	3.6% (1)	13.3% (11)	*p* = 0.152
C5		52.9% (36)	31.3% (10)	17.9% (5)	13.3% (11)	*p* = 0.28
C6		50.0% (34)	37.5% (12)	21.4% (6)	22.9% (19)	*p* = 0.156
Sural	Calf	35.3% (24)	28.1% (9)	10.7% (3)	12.0% (10)	*p* = 0.05

**Table 5:** Nerve conduction studies. One patient in the diabetes without polyneuropathy group (group 3) refused to undergo NCS. High missing numbers as indicated for the individual measurements were mainly caused by electrically not excitable nerves. A p value < 0.05 was considered statistically significant.

			Group 1 (CIDP without diabetes)	Group 2 (CIDP with diabetes)	Group 3 (Diabetes without neuropathy)	Group 4 (Diabetes with DPN)	Mann-Whitney U test group 2 vs. group 4
Measurement		Cut-off value	68	32	24	83	
Median Nerve	distal motor latency, ms (IQR)	4.2	4.1 (1.8)	4.9 (1.4)	4.1 (0.7)	4.4 (1.1)	*p* = 0.632
	CMAP amplitude, mV (IQR)	5.0	10.0 (6.1)	7.6 (4.9)	10.7 (7.0)	9.3 (4.6)	*p* = 0.280
	MCV, m/s (IQR)	50.0	46.6 (12.8)	43.6 (11.2)	52.8 (3.9)	50.0 (6.0)	*p* < 0.001
	SNAP, µV (IQR)	6.9	5.1 (8.2)	0.9 (7.7)	16.8 (17.8)	10.4 (8.9)	*p* < 0.001
	SCV, m/s (IQR)	45	45.0 (11.8) 19 (27.9%) missing	43.7 (13.3) 16 (50.0%) missing	49.1 (11.5)	42.9 (9.4) 9 (10.8%) missing	*p* = 1.000
	minimal F-Wave latency, ms (IQR)	31.0	32.8 (6.3) 14 (20.6%) missing	32.7 (6.1) 12 (37.5%) missing	26.0 (3.6)	29.2 (3.7) 2 (2.4%) missing	*p* = 0.007
Tibial Nerve	distal motor latency, ms (IQR)	5.1	5.1 (3.0)	5.4 (2.0)	4.2 (1.4)	4.7 (1.7)	*p* = 0.196
	CMAP amplitude, mV (IQR)	5.0	1.6 (5.73)	1.0 (3.7)	12.4 (9.9)	6.7 (8.2)	*p* < 0.001
	MCV, m/s (IQR)	40.6	37.0 (9.7) 12 (17.6%) missing	35.7 (6.5) 11 (34.4%) missing	42.8 (6.4)	37.9 (5.9) 7 (8.4%) missing	*p* = 0.479
	minimal F-Wave latency, ms (IQR)	58.0	67.0 (15.7) 40 (58.8%) missing	67.0 (14.6) 23 (71.9%) missing	52.8 (4.3)	59.1 (7.4) 10 (12.0%) missing	*p* = 1.000
Sural Nerve	SNAP amplitude, µV (IQR)	< 40 years: 4.9 > 40 years: 3.8	2.1 (6.3) 5 (7.4%) missing	0 (0) 4 (12.5%) missing	5.6 (4.3)	0 (2.7)	*p* = 1.000
	SCV, m/s (IQR)	< 40 years: 41.3 > 40 years: 39.3	40.0 (9.6) 33 (48.5%) missing	35.5 (5.7) 26 (81.3%) missing	42.8 (4.5) 2 (8.3%) missing	38.2 (4.2) 49 (59.0%) missing	*p* = 1.000

**Table 6:** Multiple binary logistic regression. A p value < 0.05 was considered statistically significant.

Parameter	Odds Ratio	95% Confidence interval	*p* value
UPSS	0.558	0.343–0.909	*p* = 0.019
ONLS	0.377	0.225–0.631	*p* < 0.001
Tibial nerve, distal CMAP	0.97	0.833–1.128	*p* = 0.691
Median nerve, MCV	1.113	0.989–1.251	*p* = 0.075
Median nerve, SNAP	1.096	0.936–1.282	*p* = 0.255
Median nerve F-wave latency	1.05	0.78–1.414	*p* = 0.748

## Electronic supplementary material

Supplementary Material 1

## Data Availability

The data that support the findings of this study are available on reasonable request from the corresponding author.
