# Supplementary material for: Nerve ultrasound helps to distinguish CIDP patients with diabetes from patients with diabetic polyneuropathy
Source: Sci Rep. 2024 Dec 16;14:30504. doi: 10.1038/s41598-024-82235-8 (PMC11649805; doi:10.1038/s41598-024-82235-8)
Supplement: Supplementary file 1 — Supplementary Material 1 [file 41598_2024_82235_MOESM1_ESM.docx]

| Scanned nerve segments | Anatomical location |
| --- | --- |
| Vagus Nerve | Carotid sheath at the carotid bulb |
| C5 longitudinal  Transversal process | directly after leaving the intervertebral foramen |
| C6 longitudinal  Transversal process | directly after leaving the intervertebral foramen |
| Median nerve, upper arm | mid-humerus, next to brachial artery and biceps brachii muscle |
| Median nerve, elbow | next to pronator teres muscle |
| Median nerve, forearm | mid-forearm, between superficial digital flexor and deep digital flexor muscle, 15 cm above the retinaculum flexorum |
| Median nerve, wrist | at the inlet of the carpal tunnel, at the level of the pisiform bone |
| Ulnar nerve, upper arm | in the middle of the humerus next to the medial head of triceps brachii muscle and nearby the biceps brachii muscle |
| Ulnar nerve, elbow | at ulnar sulcus |
| Ulnar nerve, forearm | mid-forearm, 15 cm above the retinaculum flexorum |
| Ulnar nerve, wrist | at the Guyon canal |
| Tibial nerve, popliteal | mid-popliteal space next to medial and lateral gastrocnemius muscle |
| Tibial nerve, ankle | next to the (mostly) 3 deep tibial veins and artery near the medial malleolus, before nerve division into plantar nerves |
| Fibular nerve, popliteal | lateral popliteal space next to the long head of biceps femoris muscle, 2 cm above fibular head |
| Fibular nerve, fibular head | at the fibular head |
| Sural nerve | at the mid-calf |

**Table S1. Anatomical locations of the scanned nerve segments.**
